# The Scientific American

**Published:** 1871-12

**Authors:** 


*The Scientific American.*

This valuable paper enters upon its twenty-seventh year with the opening of 1872.

It will continue its interesting papers and editorials on New Inventions, Architecture, Agriculture, Engineering, etc., etc., and cannot fail to be interesting to the farmer, mechanic, and in fact to all who are interested in the progress of Science and Art. It will also contain a weekly official list of all patents issued. The numbers of Scientific American are all finely illustrated and are printed on the finest quality of paper. It is a paper interesting as well in the family circle as in the work shop, and cannot fail to give satisfaction.

